# Impact of initial management on respiratory complications in emergency department patients requiring mechanical ventilation

**DOI:** 10.1186/2197-425X-3-S1-A670

**Published:** 2015-10-01

**Authors:** SJ Lee, YR Jung, MK Lee, SH Kim, SJ Yong, WY Lee

**Affiliations:** Yonsei University Wonju College of Medicine, Internal Medicine, Wonju, Korea, Republic of

## Background

Outcomes of patients with acute respiratory distress syndrome (ARDS) show a trend of improvement with the lung protective strategy. Recently, the focus turns to how to prevent ARDS in critically ill patients. Especially in emergency department (ED), the early treatment plan influences the following management in intensive care unit (ICU).

## Objectives

We designed this study to investigate risk factors for respiratory complications including ARDS in patients without lung injury who admitted ED and required mechanical ventilation (MV).

## Methods

Following a retrospective review of clinical data and radiographic findings of the patients admitted to the ED from April, 2014 to December, 2014, 100 patients who required MV for non-thoracic cause and were under MV care in non-medical ICU at least during one day. Development of respiratory outcomes including ARDS during one week after admission was described and the risk factors for respiratory complications were analyzed.

## Results

The median age of the patients was 64 year (21 - 99) and 41% (n=41) was female. Of total 100 Patients requiring invasive MV, 43 cases (43%) were drug or chemical intoxication. Neurologically complicated patients and trauma patients were accounted for 41% and 16%, respectively. Mean tidal volume and PEEP in initial ventilator mode was 7.30 ± 1.93 mL/kg (predicted body weight, PBW) and 6.02 ± 0.97 cmH_2_O. Mean tidal volume of initial ventilator mode with and without respiratory complications was 7.61 ± 1.49 mL/kg and 7.12 ± 2.14 mL/kg (p = 0.24). Respiratory complications developed in 32 patients (32%). Among 32 patients, four patients underwent ARDS. Other respiratory complications included pneumonia (n=25, 25%), pulmonary embolism (n=2, 2%), and atelectasis (n=11, 11%). In-hospital mortality developed in 12 patients (12%). The factors associated with respiratory complications by univariate analysis (*p* < 0.2) were large volume of fluid, large amount of transfusion, and requiring inotrope. After adjusting for age and sex, multiple logistic regression analysis with variables including inotrope, tidal volume per PBW, transfusion amount, and volume of fluid revealed that only whether the patients required inotrope or not (OR 3.66, 95% CI 1.24 - 10.80, *p* = 0.019) influenced the results of respiratory complications.

## Conclusions

Requiring inotropes during care in ED was an independent risk factor for respiratory complications including ARDS, while tidal volume was not associated with the outcomes. This finding might be resulted from routine apply of low tidal volume in most patients in ED of our hospital. Further studies suggesting most proper ventilator setting will be needed.

